# Flaxseed Oil Containing ***α***-Linolenic Acid Ester of Plant Sterol Improved Atherosclerosis in ApoE Deficient Mice

**DOI:** 10.1155/2015/958217

**Published:** 2015-06-09

**Authors:** Hao Han, Peipei Yan, Li Chen, Cheng Luo, Hui Gao, Qianchun Deng, Mingming Zheng, Yong Shi, Liegang Liu

**Affiliations:** ^1^Department of Nutrition and Food Hygiene, Hubei Key Laboratory of Food Nutrition and Safety, School of Public Health, Tongji Medical College, Huazhong University of Science and Technology, Wuhan 430030, China; ^2^Key Laboratory of Environment and Health, Ministry of Education & Ministry of Environmental Protection and State Key Laboratory of Environmental Health (Incubating), School of Public Health, Tongji Medical College, Huazhong University of Science and Technology, Wuhan 430030, China; ^3^Oil Crops Research Institute, Chinese Academy of Agricultural Sciences, Hubei Key Laboratory of Oil Crops Lipid Chemistry and Nutrition, Hubei University of Technology, Wuhan 430030, China; ^4^School of Food and Pharmaceutical Engineering, Hubei University of Technology, Wuhan 430030, China

## Abstract

Plant sterols (PS) have potential preventive function in atherosclerosis due to their cholesterol-lowering ability. Dietary *α*-linolenic acid in flaxseed oil is associated with a reduction in cardiovascular events through its hypolipidemic and anti-inflammation properties. This study was designed to evaluate the effects of flaxseed oil containing *α*-linolenic acid ester of PS (ALA-PS) on atherosclerosis and investigate the underlying mechanisms. C57BL/6 mice were administered a regular diet and apoE knockout (apoE-KO) mice were given a high fat diet alone or supplemented with 5% flaxseed oil with or without 3.3% ALA-PS for 18 weeks. Results demonstrated that flaxseed oil containing ALA-PS was synergistically interaction in ameliorating atherosclerosis as well as optimizing overall lipid levels, inhibiting inflammation and reducing oxidative stress. These data were associated with the modification effects on expression levels of genes involved in lipid metabolism (PPAR*α*, HMGCR, and SREBPs), inflammation (IL-6, TNF, MCP-1, and VCAM-1), and oxidative stress (NADPH oxidase).

## 1. Introduction

Atherosclerosis is the primary cause of cardiovascular diseases (CVD), which is one of the leading causes of mortality and morbidity in the world [1]. Although the precise mechanisms for atherosclerosis have not been fully elucidated, an elevated concentration of low-density lipoprotein cholesterol (LDL-C) is a well-established independent risk factor for atherosclerosis [2]. In addition, LDL oxidation has been illustrated as one of the initial steps of atherogenesis [3]. Therefore, dietary and pharmacologic interventions targeted at lowering LDL-C are very important elements for atherosclerosis prevention.

The pathophysiological significance of plant sterols (PS), one kind of natural compounds presenting in plant oils, nuts, cereals, and legumes, has been intensively discussed in previous reports. It has been reported that consumption of 2 g/day PS could reduce LDL-C by 10–15% in about 90% of individuals [4]. Another previous publication also demonstrated that supplementation of PS in diet may have decreased LDL-C level (10%) for reducing risk of cardiovascular (10–20%) [5]. However, PS was not observed to have any beneficial effect on triacylglycerol (TG) and high-density lipoprotein cholesterol (HDL-C) [6], two important independent factors for cardiovascular disease [7, 8]. In addition, purified PS was shown to have low intestinal bioavailability and poor solubility in diet. Because n-3 polyunsaturated fatty acids (PUFAs) have potent hypotriglyceridemic and HDL-C elevation effects, PS has been recently esterified by fish oil fatty acids to obtain final product plant sterols esters which have overall beneficial effects on lipids [9], well bioavailability, and extensive solubility in fat. Therefore, plant sterols esters can be incorporated into fatty foods, such as margarines, yoghurt, and spreads [10]. However, fish oil fatty acid is insufficient supply nutrient, and margarines, yoghurt, and spreads are not the main food in China. *α*-Linolenic acid (ALA, C18:3n-3), the major n-3 fatty acid, widely exists in flaxseed oil which is a traditional edible oil in China. As the precursor of longer chain n-3 PUFA (EPA and DHA), ALA had good effects on TG reduction and HDL-C elevation [11]. More importantly, a large number of epidemiological evidences showed that consumption of ALA could protect against CVD [12, 13]. These data suggest that ALA may be a perfect vehicle for PS esterification and the combination treatment may synergistically improve atherosclerosis. Meanwhile, as one of the high frequency and largely consumed oils in Chinese daily cooking, flaxseed oil is a good way for Chinese to get enough PS. However, the efficiency of this novel flaxseed oil fortified with *α*-linolenic acid ester of plant sterol (ALA-PS) has not been shown.

In the current study, we detected the effects of dietary flaxseed oil containing ALA-PS on atherosclerosis in apoE-KO mice and investigated the underlying molecular mechanisms.

## 2. Materials and Methods

### 2.1. Study Design

A total of 42 male apoE-KO mice and 14 wild type C57BL/6 mice (6 weeks of age, 15–19 g) were purchased from Peking University Resources Centre (Permission number: SCXK 2011-0012). After one-week acclimatization period, 14 wild type C57BL/6 mice were assigned to control group and given normal chow. 42 male apoE-KO mice were randomly divided into HFD-, FO-, and FO+ALA-PS-treated groups matched with their mean body weight and plasma total cholesterol levels as previously published methods [14]. HFD group was given a high fat diet containing of 21% fat and 0.15% cholesterol [15]. This high fat diet was further supplemented with 5% (w/w) flaxseed oil for FO group; a combination of 3.3% (w/w) ALA-PS (provided 2% PS) mixture with flaxseed oil which provided equivalent ALA to FO group was added to the high fat diet and used for ALA-PS group. The flaxseed oil was manufactured by Inner Mongolia Caoyuan Kanghen Food Co. Ltd. (Hohhot, China) and contained 57.82% ALA (% of total fatty acids). Plant sterol (*β*-sitosterol 77%, campesterol 17%, and stigmasterol 5%) was provided by BASF Co. Ltd. (Shanghai, China). ALA (*α*-linolenic acid 80%, linoleic acid 15%, and oleic acid 5%) was purchased from Henan Linuo Biochemical Co. Ltd. (Anyang, Henan, China). The ALA-PS was synthesized by Oil Crops Research Institute, Chinese Academy of Agricultural Sciences, Wuhan, China (Wuhan, China) using PS and ALA mentioned above [16]. The animals were maintained in constant temperature-controlled rooms (25 ± 2°C) with controlled lighting (12 h light-dark cycles). The investigation conformed to the Guide for the Care and Use of Laboratory Animals published by the US National Institutes of Health and was approved by Tongji Medical College Council on Animal Care Committee.

After 18 weeks, three animals in each group were randomly selected for en face aorta and aortic sinus Oil-Red O staining to observe their pathological changes and lipid deposition. The remaining animals were feed-deprived for 8 h, anesthetized with ketamine HCl (50 mg/kg)/xylazine (10 mg/kg), and subsequently killed by cervical dislocation. Blood was collected and centrifuged for 20 min at 5000 g at 4°C and then stored at −80°C. Aorta and liver were excised, immediately frozen in liquid nitrogen, and stored at −80°C until use.

### 2.2. Histology and Morphometry Evaluations of Atherosclerotic Lesions

For en face analysis, the aorta from the ascending arch to the iliac bifurcation was cleaned of peripheral tissue, opened longitudinally, pinned flat, and stained with Oil-Red O. Images were captured with Canon IXUS 220 HS camera.

The heart samples were embedded in tissue freezing medium O.C.T. and sectioned into consecutive 8 *μ*m thick sections at −20°C. The distal end of the aortic sinus was recognized by the disappearance of the three aortic valve cusps. Every sixth section was stained with Oil-Red O and digitally photographed under magnification (×40).

Images were analyzed using Image-Pro Plus (IPP) software. The lesion area index was calculated as the percentage of aortic lumen area covered by atherosclerotic lesions.

### 2.3. Immunohistochemistry Assay

Aortic arch cross-sections were immunostained with CD68 (1 : 400; ab955, Abcam Limited), Ly-6C (1 : 200; sc-52650, Santa Cruz Biotechnology), and *α*-smooth muscle actin antibodies (1 : 500, A 5228, Sigma-Aldrich) to detect macrophages, Ly-6C-positive macrophages, and smooth muscle cells. After washing, sections were incubated with anti-rat and anti-mouse horseradish peroxidase-conjugated secondary antibodies at 1 : 200 dilutions (CST). Immunocomplexes containing CD68, Ly-6C, and *α*-smooth muscle actin antibodies were detected using diaminobenzidine tetrahydrochloride dihydrate substrate (DAB; Sigma). The sections were digitally photographed under magnification (×200).

### 2.4. Determination of Lipid Parameters in Serum and Liver

The concentrations of TC, TG, LDL-C, and HDL-C in serum and liver were measured by enzymatic colorimetric assays using commercially available detection kits (Biosino Biotechnology Co., Ltd., Beijing, China).

### 2.5. Measurement of Inflammation Cytokines in Plasma

The levels of interleukin-6 (IL-6), interleukin-10 (IL-10), tumor necrosis factor alpha (TNF-*α*), monocyte chemoattractant protein-1 (MCP-1), and soluble vascular cell adhesion molecule-1 (sVCAM-1) in plasma were measured by enzyme linked immunosorbent assays using commercially available detection kits (R&D Systems, USA.).

### 2.6. Determination of Oxidative Stress Parameters in Serum and Liver

The concentrations of glutathione (GSH) were measured according to its reaction with 5,5′-dithiobis-2-nitrobenzoic acid (DTNB) into 2-nitro-5-thiobenzoic acid (TNB), following deproteinization by 5% trichloroacetic acid [17]. Malondialdehyde (MDA), an index of lipid peroxidation, was measured by using thiobarbituric acid colorimetry slightly modified by Ohkawa et al. [18]. The contents of GSH and MDA in liver were standardized by protein concentration measured by the Bio-Rad Protein Assay Kit (USA).

### 2.7. Evaluation of ROS Level in Aorta of Mice

Dihydroethidium (DHE) (Molecular Probes, Eugene, OR, USA) was used for in situ detection of reactive oxygen species (ROS) in the aorta of mice. Fresh cross-sections (5 *μ*m) of unfixed but frozen aorta were immediately incubated with 5 M DHE at 37°C for 15 min in a humidified chamber. Fluorescence level was then visualized with a fluorescence microscope. Fluorescence intensities in randomly selected areas of the images were quantified by using the IPP image analysis software.

### 2.8. Real-Time RT-PCR Analysis

Total RNA was isolated from the stored frozen liver, aorta tissue, and fresh blood monocytes using the Trizol reagent (Invitrogen, 154 Carlsbad, CA, USA). Messenger RNA (mRNA) expression was quantified by using specific oligo primers and SYBRGreen-based qRT-PCR kit (TaKaRa Biotechnology Co., Ltd., Dalian, China) in 7900HT instrument (Applied Biosystems, Forster, CA, USA). The specificity of the product was assessed from melting curve analysis. Gene expressions were determined using the 2^−ΔΔCt^ method. The mRNA of *β*-actin was quantified as an endogenous control. Gene expressions are presented as fold change relative to control. Quantitative real-time PCR primers were as shown in Table 1.

### 2.9. Western Blot Analysis

Thoracic aorta, liver tissues, and blood monocytes were homogenized and lysed in RIPA lysis buffer (1% Triton X-100, 1% deoxycholate, and 0.1% SDS). Tissue lysates with equal protein amounts were subjected to western blotting. The protein was separated by 10% SDS-polyacrylamide gel and then transferred onto a PVDF membrane. The membranes were incubated with specific primary antibodies under overnight at 4°C after blocked with 5% nonfat milk solution. Then the target proteins were incubated with the species-specific secondary antibodies conjugated to horseradish peroxidase. Immunoreactive bands were detected by means of an ECL plus Western Blotting Detection System (Amersham Biosciences, Little Chalfont, UK) according to the manufacturer's instructions. Quantitative analysis of the relative density of the bands in western blots was performed by Quantity One 4.62 software (Bio-Rad, Hercules, CA, USA). Data were corrected for background standardized to *β*-actin as optical density (OD/mm^2^). Primary antibodies were as follows: monoclonal anti-*β*-actin (Sigma), rabbit anti-SREBP2 (Abcam Limited), rabbit antibody anti-SREBP1 (Abcam Limited), rabbit anti-PPAR*α* (Abcam Limited), rabbit anti-HMGCr (Abcam Limited), rabbit anti-IL-6 (Cell Signaling Technology), mouse anti-TNF alpha (Abcam Limited), rabbit anti-VCAM1 (Abcam Limited), rabbit anti-MCP1 (Abcam Limited), mouse anti-IL-1*β* (Cell Signaling Technology), rabbit anti-p22-phox (Santa Cruz), rabbit anti-p47-phox (Merck Millipore Corporation), rabbit anti-p67-phox (Merck Millipore Corporation), rabbit anti-gp91-phox (Abcam Limited), anti-rabbit IgG HRP-linked antibody (Cell Signaling Technology), and anti-mouse IgG HRP-linked antibody (Cell Signaling Technology).

### 2.10. Statistical Analysis

Results are expressed as means ± SD, and *P* < 0.05 was considered significant. Statistical analyses of data were performed using one-way analysis of variance with SPSS 12.0 software package (SN: 59245 46841 40655 89389 09859 21671 21957 29589 12).

## 3. Results

### 3.1. FO+ALA-PS Ameliorated Atherosclerosis Induced by HFD in Mice

**Figure 1:**
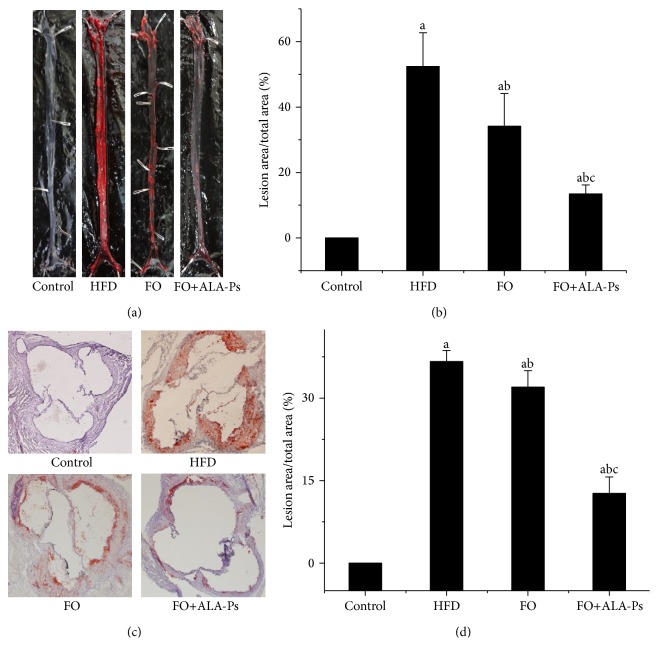
Intervention of FO+ALA-PS ameliorated atherosclerosis in HFD diet-fed mice. Photomicrographs of representative Oil-Red O stained aortas of mice in control, HFD, FO, and ALA-PS groups (a). Quantitative analysis of the atherosclerotic lesion aortas of mice. The lesion area was measured as the percentage of aortic luminal area covered by atherosclerotic lesions. Values are given as mean ± standard deviation of the mean (*n* = 3). ^a^ *P* < 0.05 versus the control; ^b^ *P* < 0.05 versus the HFD group; ^c^ *P* < 0.05 versus the FO group (b). Representative images of cross-sections taken of the aortic sinuses obtained from mice in control, HFD, FO, and ALA-PS groups. The sections of aortic sinuses were stained with Oil-Red O (magnification ×40) for lipid deposition (red) and cross-stained with hematoxylin (blue) (c). Quantitative analysis of the atherosclerotic lesions in aortic sinuses of mice. The lesion area was measured as the percentage of aortic luminal area covered by atherosclerotic lesions. Values are given as mean ± standard deviation of the mean (*n* = 3). ^a^ *P* < 0.05 versus the control; ^b^ *P* < 0.05 versus the HFD group; ^c^ *P* < 0.05 versus the FO group (d).

En face analysis of aorta revealed atherosclerotic lesion formation with the aid of Oil-Red O staining. As representative results showed in Figure 1(a), compared with control, a significantly atherogenic action was induced in apoE-KO mice given HFD while the lesions in the FO and FO+ALA-PS groups were obviously decreased. Quantitative analysis of the atherosclerotic lesions in the aortas of mice (Figure 1(b)) demonstrated statistically smaller lesion size in FO+ALA-PS-treated animals than those in FO-treated mice (13.43 ± 2.75% versus 34.13 ± 10.00%).

Cross-sectional analysis of atherosclerotic development at the aortic sinus revealed lipid deposition with the aid of Oil-Red O staining. Representative images were shown in Figure 1(c). The control group was shown to have little lipid deposition. Atherosclerotic lesions at the aortic sinus were extensive following HFD feeding, while FO and FO+ALA-PS treatment groups had much less lipid deposition. The extent of atherosclerotic development at the aortic sinus was quantified as a percentage of aortic cross-sectional luminal area occupied by Oil-Red O-stained lipid deposits. As illustrated in Figure 1(d), the addition of flaxseed oil to an atherogenic diet significantly inhibited the development of atherosclerotic lesions at the aortic sinus, and a further improvement effect was observed in FO+ALA-PS intervention group (12.67 ± 2.08% versus 32.00 ± 2.65%). These results indicated that the addition of ALA-PS markedly strengthened the antiatherogenic properties of flaxseed oil in apoE-KO mice.

### 3.2. Anti-Inflammatory and Antiproliferative Actions of FO+ALA-PS

**Figure 2:**
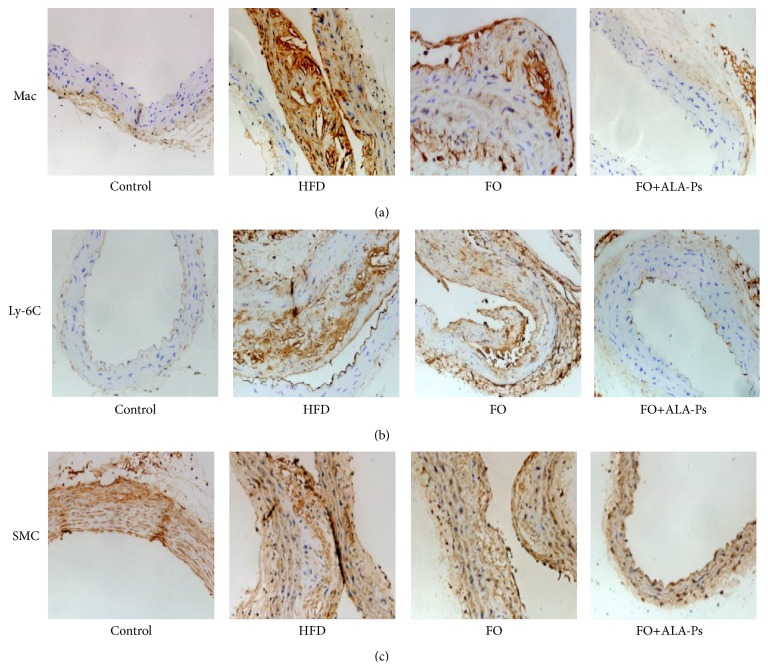
Treatment of FO+ALA-PS prevents atherosclerotic development via anti-inflammatory and antiproliferative actions. Representative images of aortic cross-sections immunostained with markers of macrophage infiltration, inflammation, and proliferation from mice fed a normal, HFD, FO, or ALA-PS diet for 18 wk. Immunoreactivity to MAC (a), Ly-6C (b), and SMC (c) antibodies are evident with brownish diaminobenzidine staining (magnification ×400).

Inflammation is now considered to be an important mechanistic process involved in atherosclerosis, and markers of inflammation are also detected as a function of the dietary interventions. The macrophage marker CD68 and Ly-6C have been used as an indicator of inflammatory reactions associated with atherosclerosis. As shown in Figures 2(a) and 2(b), mice fed HFD were characterized by increased infiltration of macrophages and Ly-6C monocyte compared with control. Flaxseed oil supplementation alone did not reduce macrophages, but when included with ALA-PS, flaxseed oil was capable of mitigating the accumulation of macrophages.

In addition, atherosclerotic plaque is generated partly by proliferation and migration of vascular smooth muscle cells (VSMC), and *α*-actin can be used as a marker of VSMC proliferation within the vessel wall. As shown in Figure 2(c), intimal VSMC contents were pronounced in mice of HFD and FO groups while dietary FO+ALA-PS inhibited proliferation and migration of VSMC.

### 3.3. Combined Treatment with FO+ALA-PS Improved Lipid Metabolism

**Figure 3:**
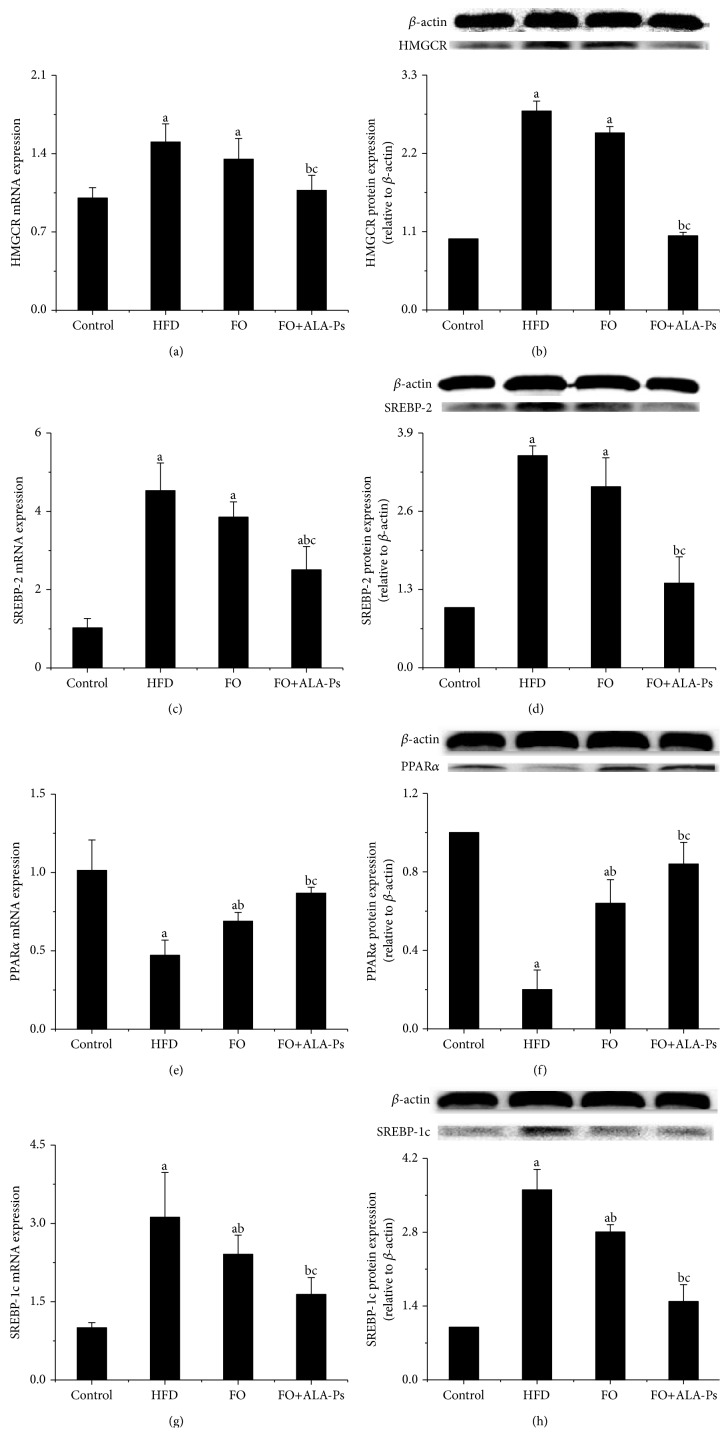
Effects of FO+ALA-PS on mRNA and protein expressions of hepatic HMGCR (a-b), SREBP-2 (c-d), PPAR*α* (e-f), and SREBP-1c (g-h) in mice. After intervention by flaxseed oil with ALA-PS for 18 weeks, total RNA was extracted from liver of mice by Trizol. HMGCR, SREBP-2 PPAR*α*, and SREBP-1c mRNA expressions were analyzed by real-time RT-PCR. The mRNA of *β*-actin was quantified as an endogenous control. Hepatic lysates were prepared and immunoblotted with corresponding antibody, respectively. Blotting with anti-*β*-actin was used as a protein loading control. HMGCR, SREBP-2, PPAR*α*, and SREBP-1c are presented as fold change relative to control. Representative immunoblots are shown. Values are given as mean ± standard deviation of the mean (*n* = 3). ^a^ *P* < 0.05 versus the control; ^b^ *P* < 0.05 versus the HFD group; ^c^ *P* < 0.05 versus the FO group.

The intervention of flaxseed oil alleviated the rise of TG induced by HFD in serum and liver while a much larger decrease in TG was observed in FO+ALA-PS group. Although flaxseed oil feeding alone elevated serum HDL-C, it had no effect on LDL-C and TC. Combination treatment not only apparently reduced LDL-C and TC but also increased the concentration of HDL-C (Table 2). To investigate the underlying molecular mechanism by which dietary FO+ALA-PS modulates lipid metabolism, gene and protein expressions of the major factors involved in hepatic cholesterol homoeostasis, fatty acid catabolism, and synthesis were detected. As illustrated in Figure 3, mRNA and protein expressions of 3-hydroxy-3-methylglutaryl-CoA reductase (HMGCR) and sterol regulatory element binding protein 2 (SREBP-2) were found to be statistically decreased in FO+ALA-PS-treated but not FO-treated animals, as compared to those in HFD-fed animals. In addition, flaxseed oil intervention elevated peroxisome proliferator-activated receptor *α* (PPAR*α*) and reduced sterol regulatory element-binding protein 1c (SREBP-1c), respectively. A more pronounced effect was achieved by FO+ALA-PS application.

### 3.4. FO+ALA-PS Attenuated Inflammation

**Figure 4:**
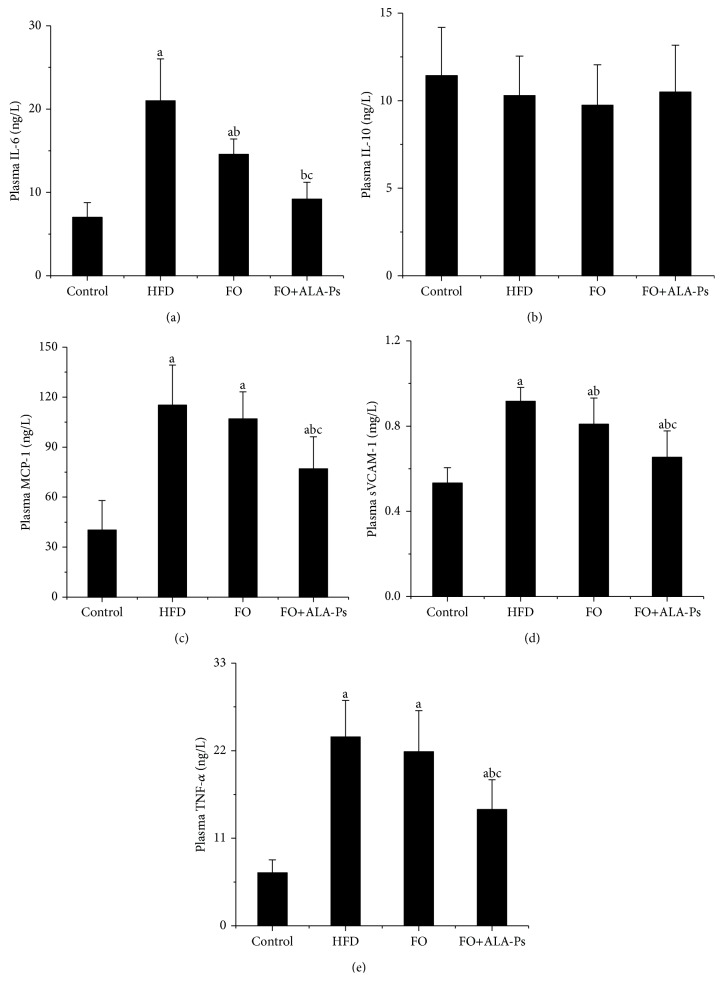
Supplementation of FO+ALA-PS reduced plasma inflammatory cytokines in mice. Values are means for plasma levels of IL-6 (a), IL-10 (b), MCP-1 (c), sVCAM-1 (d), and TNF-*α* (e). Values are given as mean ± standard deviation of the mean (*n* = 14). ^a^ *P* < 0.05 versus the control; ^b^ *P* < 0.05 versus the HFD group; ^c^ *P* < 0.05 versus the FO group.

**Figure 5:**
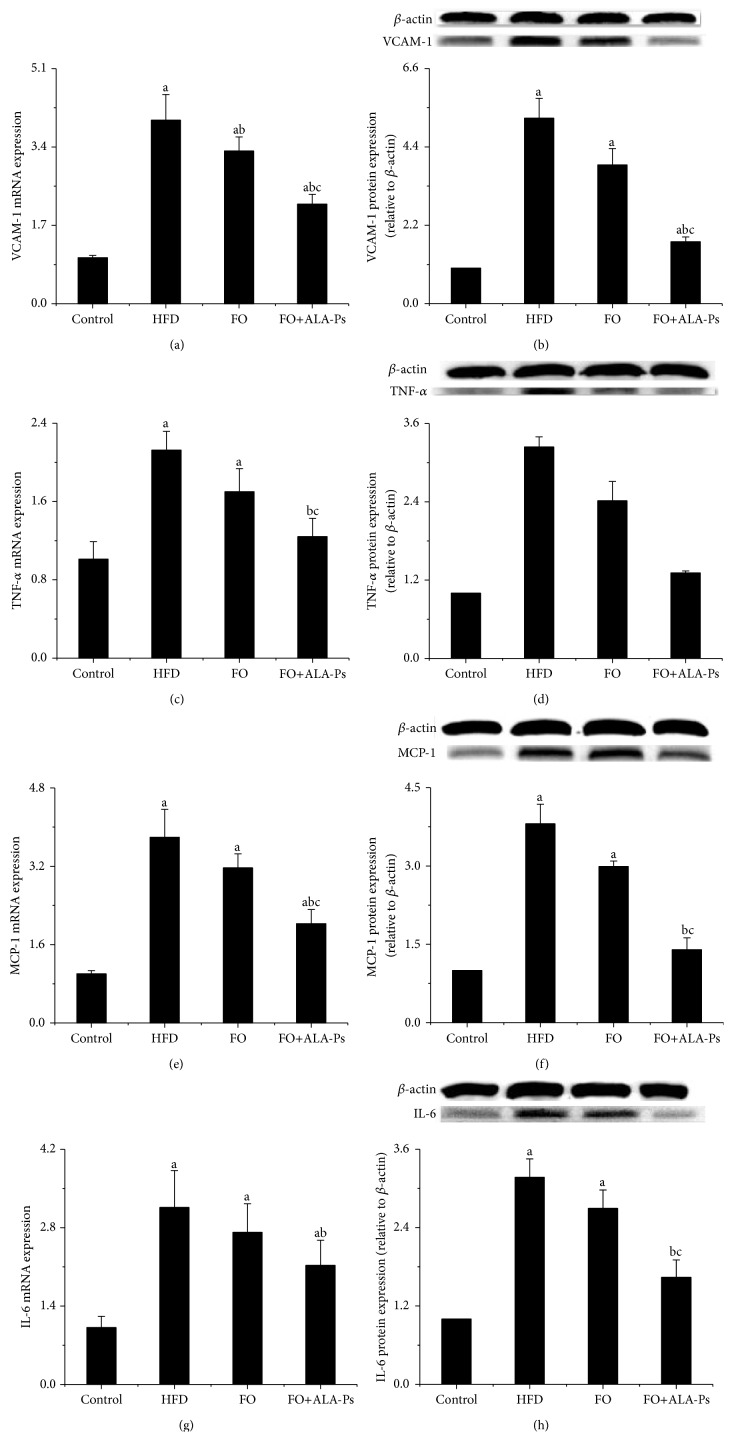
Effects of FO+ALA-PS on mRNA and protein expressions of aortic VCAM-1 (a-b), TNF-*α* (c-d), MCP-1 (e-f), and IL-6 (g-h) in mice. After combining treated flaxseed oil with ALA-PS for 18 weeks, total RNA was extracted from aorta of mice by Trizol. VCAM-1, TNF-*α*, MCP-1, and IL-6 mRNA expressions were analyzed by real-time RT-PCR. The mRNA of *β*-actin was quantified as an endogenous control. Aortic lysates were prepared and immunoblotted with corresponding antibody, respectively. Blotting with anti-*β*-actin was used as a protein loading control. VCAM-1, TNF-*α*, MCP-1, and IL-6 are presented as fold change relative to Control. Representative immunoblots are shown. Values are given as mean ± standard deviation of the mean (*n* = 3). ^a^ *P* < 0.05 versus the control; ^b^ *P* < 0.05 versus the HFD group; ^c^ *P* < 0.05 versus the FO group.

**Figure 6:**
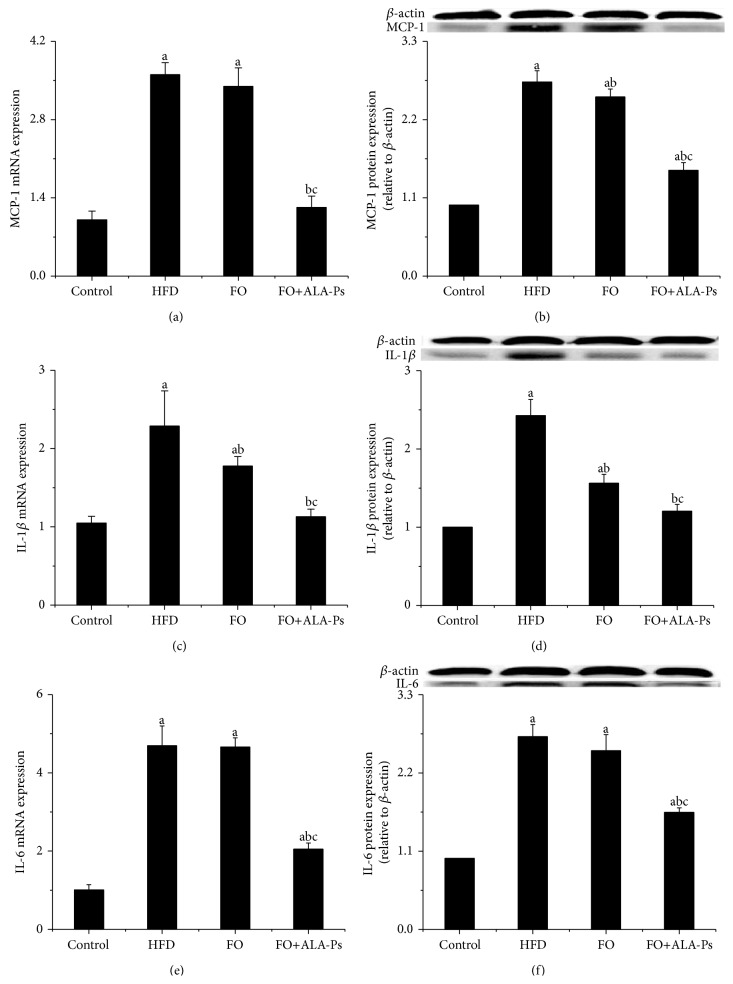
Effects of FO+ALA-PS on mRNA and protein expressions of MCP-1 (a-b), IL-1*β* (c-d), and IL-6 (e-f) in circulating monocytes of mice. After supplementing flaxseed oil with ALA-PS for 18 weeks, total RNA was extracted from circulating monocytes of mice by Trizol. MCP-1, IL-1*β*, and IL-6 mRNA expressions were analyzed by real-time RT-PCR. The mRNA of *β*-actin was quantified as an endogenous control. Monocytes lysates were prepared and immunoblotted with corresponding antibody, respectively. Blotting with anti-*β*-actin was used as a protein loading control. MCP-1, IL-1*β*, and IL-6 are presented as fold change relative to control. Representative immunoblots are shown. Values are given as mean ± standard deviation of the mean (*n* = 3). ^a^ *P* < 0.05 versus the control; ^b^ *P* < 0.05 versus the HFD group; ^c^ *P* < 0.05 versus the FO group.

Compared to HFD, mice administrated by flaxseed oil had 30.6% and 11.9% less plasma IL-6 and VCAM-1, while this decrease was 56.3% and 29.3% in mice given FO+ALA-PS. In addition, FO+ALA-PS had a significant decline effect on plasma sVCAM-1, TNF-*α*, and MCP-1 which were initially increased by HFD but not diminished by flaxseed oil intervention. However, there were no significant differences observed in plasma IL-10 in the four groups (Figure 4). On molecular level, we compared the ability of FO and FO+ALA-PS treatment in modulating aortic inflammation indicators. As shown in Figure 5, FO+ALA-PS was more effective than FO in decreasing mRNA and protein expressions of aortic VCAM-1, TNF-*α*, MCP-1, and IL-6 elevated by HFD. In an effort to further investigate molecular pathways involved in inflammation induction by HFD and the concomitant protection exerted by FO+ALA-PS on this process, we also examined the impact of FO+ALA-PS on the key inflammatory cytokines in circulating monocytes. The results clearly indicated that MCP-1, interleukin-1 beta (IL-1*β*), and IL-6 were upregulated in response to HFD, while such an effect was almost completely counteracted by the FO+ALA-PS supplement. However, flaxseed oil added alone only significantly modified the mRNA and protein expression of IL-1*β* (Figure 6).

### 3.5. Dietary FO+ALA-PS Inhibited Oxidative Stress

**Figure 7:**
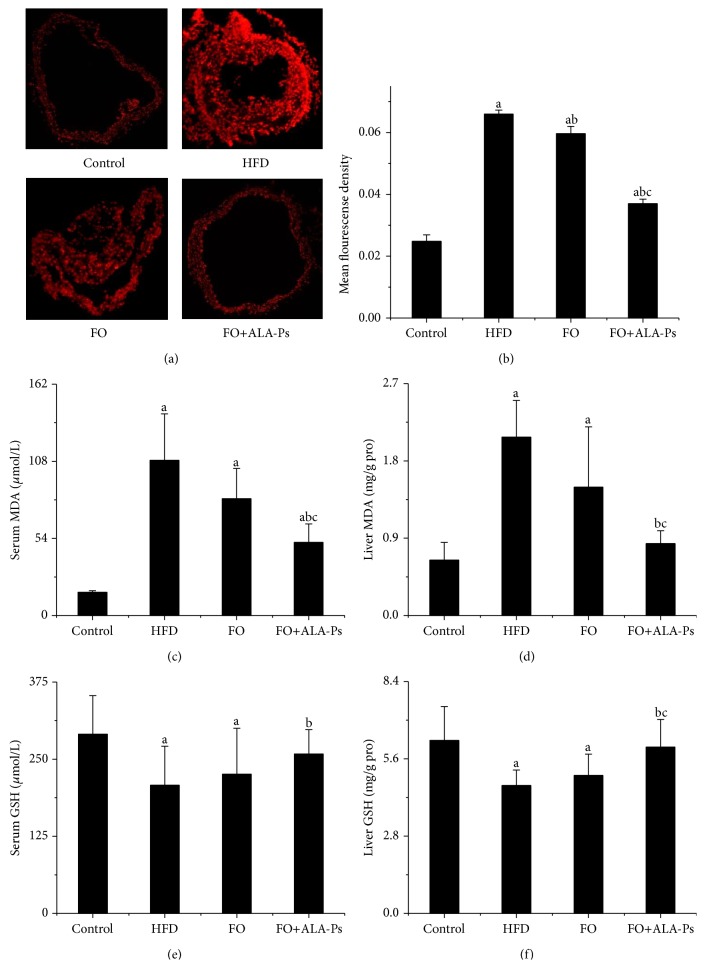
Effects of FO+ALA-PS on aortic ROS production and the levels of MDA and GSH in serum and liver of mice. ROS in the aorta of the mice was detected by using DHE which reacts with ROS and forms ETH that binds to DNA and produces red fluorescence signal, visualized with fluorescence microscope (×200) and quantified (a). Fluorescence intensities in randomly selected areas of the images were quantified by using the IPP image analysis software. Values are given as mean ± standard deviation of the mean (*n* = 3) (b). Values are means for serum MDA level (c), liver MDA level (d), serum GSH level (e), and liver GSH level (f) (*n* = 14) with standard deviations represented by vertical bars. ^a^ *P* < 0.05 versus the control; ^b^ *P* < 0.05 versus the HFD group; ^c^ *P* < 0.05 versus the FO group.

**Figure 8:**
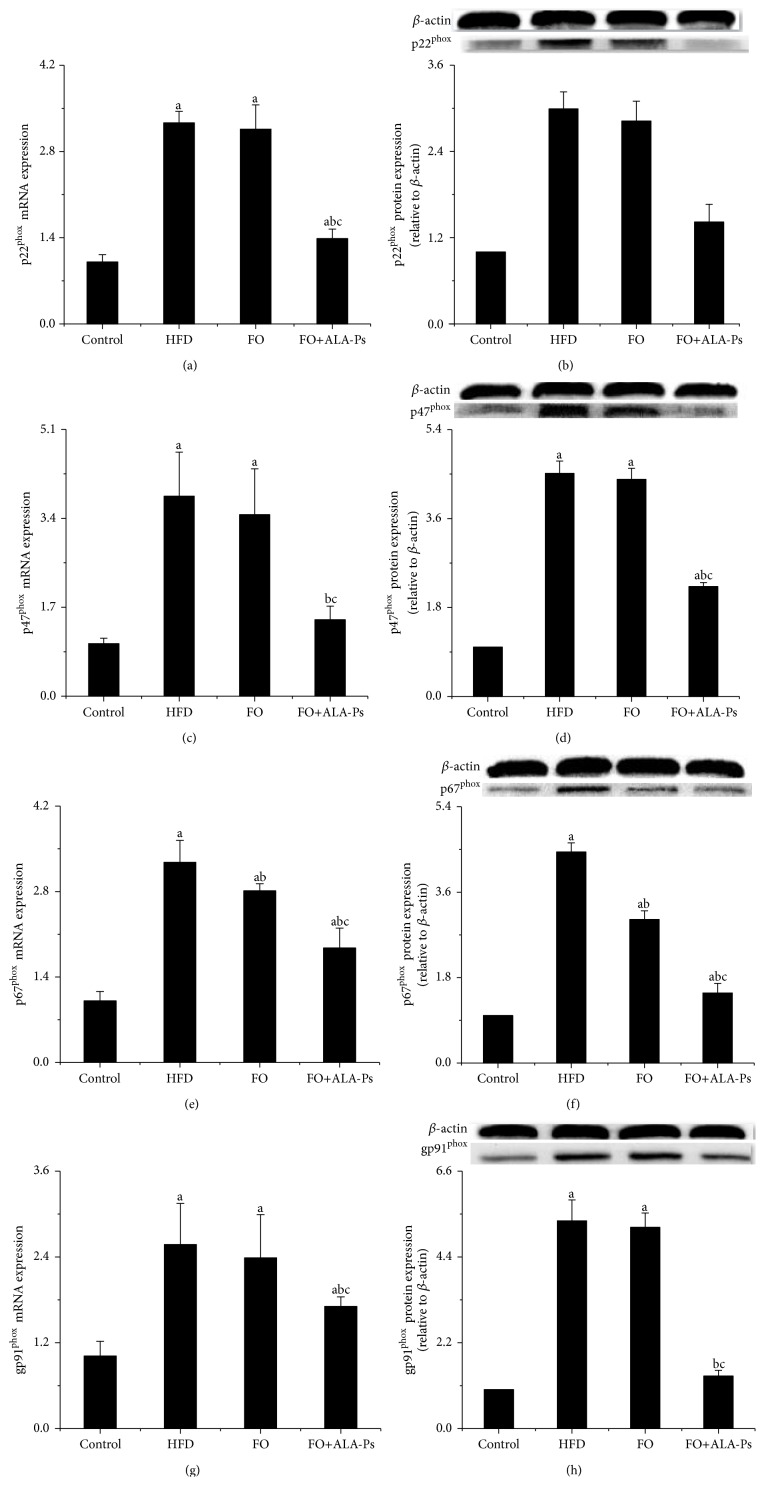
Effects of FO+ALA-PS on mRNA and protein expressions of p22^phox^ (a-b), p47^phox^ (c-d), p67^phox^ (e-f), and gp91^phox^ (g-h) in aorta of mice. Total RNA was extracted from aortas of mice by Trizol. p22^phox^, p47^phox^, p67^phox^, and gp91^phox^ mRNA expressions were analyzed by real-time RT-PCR. The mRNA of *β*-actin was quantified as an endogenous control. Aortic lysates were prepared and immunoblotted with corresponding antibody, respectively. Blotting with anti-*β*-actin was used as a protein loading control. p22phox, p47phox, p67phox, and gp91phox are presented as fold change relative to control. Values are given as mean ± standard deviation of the mean (*n* = 3). ^a^ *P* < 0.05 versus the control; ^b^ *P* < 0.05 versus the HFD group; ^c^ *P* < 0.05 versus the FO group.

Recently acquired evidence pointed out that oxidative stress is a primary contributor to atherosclerosis. In this experiment, in situ ROS production of aorta was determined. As illustrated in Figures 7(a) and 7(b), high level of ROS in aorta induced by HFD was significantly reduced after exposed to flaxseed oil, and this depression effect was further improved by adding ALA-PS. Moreover, FO+ALA-PS but not FO consumption apparently decreased the concentrations of MDA and elevated the levels of GSH in serum and liver (Figures 7(c)–7(f)). To examine the potential molecular signaling pathways of FO+ALA-PS against oxidative stress, the main subunits of nicotinamide-adenine dinucleotide phosphate (NADPH) oxidase contributing to atherosclerosis were detected. In mice given FO+ALA-PS supplemented diet, mRNA and protein expressions of aortic p22^phox^, p47^phox^, p67^phox^, and gp91^phox^ were apparently less than those of HFD-fed mice (Figure 8).

## 4. Discussion

Atherosclerotic heart disease prevention is largely attributable to factors that can be altered or prevented by lifestyle modification such as nutritional interventions, cessation of smoking, and regular exercise [19]. Recently, dietary intake of naturally occurring PS has been demonstrated to associate with a lower risk of CVD [20]. Since cardiovascular risk factors such as dyslipidemia, inflammation, and oxidative stress rarely occur in isolation, combined nutritional interventions such as n-3 PUFA and PS may provide greater risk reduction compared to either of the supplements alone. In present study, we observed that FO+ALA-PS exerted better alleviative effect on atherosclerosis in apoE KO mice than single treatment of flaxseed oil, and the underlying molecular mechanisms were related to lipid metabolism, inflammation, and oxidative stress improvement.

Hyperlipidemia is a highly predisposing condition for arteriosclerosis and other cardiovascular diseases [21]. In this study, combined supplementation with flaxseed oil and ALA-PS exhibited synergistic and complementary lipid-lowering effects. These data are supported by other studies showing overall lipid improvement effects of flaxseed oil fortified with phytosterols in rats [22]. To investigate the possible mechanisms by which FO+ALA-PS exerts its hypocholesterolemic and hypotriglyceridemic effects, the key molecules involved in lipid metabolism pathways and atherosclerosis formation were detected in this study.

SREBP-2 and HMGCR play a pivotal role in cholesterol homoeostasis. In detail, transcriptional activity of SREBP-2, a transcription factors governed cholesterol synthesis, is primarily regulated at the posttranscriptional level involving INSIG and SREBP cleavage-activating protein (SCAP). SCAP/SREBP-2 precursor complex retains in the endoplasmic reticulum via sterol-induced interaction of SCAP with INSIG [23]. When cellular cholesterol is of low level, disassociation of SCAP from INSIG frees SCAP/SREBP-2 to the Golgi, where mature SREBP-2 is released by 2-step proteolytic cleavages [24]. Then, the mature SREBP-2 enters the nucleus to induce transcription of its target genes such as HMGCR which mediates the rate-limiting step in cholesterol synthesis [25]. It has been published that dietary *β*-sitosterol and its oxidation products (SiOP) decreased mRNA expressions of SREBP-2 and HMGCR in hamster given high cholesterol diet [26]. Consistent with this result, in present study, lower mRNA and mature protein expressions of HMGCR and SREBP-2 were observed in FO+ALA-PS group but not in FO group. The FO+ALA-PS supplementation may increase the regulatory cholesterol pool and inhibit the SREBP-2 pathway to downregulate HMGCR expression, which in turn reduce the cholesterol synthesis.

In addition, previous publications illustrated that PPAR*α* and SREBP-1c were important regulators of lipogenic genes in the liver [27, 28]. It has been considered that n-3 PUFA exhibited TG-reducing effects through regulation of PPAR*α* and SREBP-1c, which control hepatic fatty acid (FA) catabolism and synthesis, respectively [29, 30]. Recently Devarshi et al. demonstrated that flaxseed oil diet improved lipid metabolism through upregulating PPAR*α* and downregulating SREBP-1 in s diabetic rats [31]. Meanwhile, aloe vera phytosterols were observed to improve the expression levels of PPAR target genes in the livers of mice with diet-induced obesity [32] and ameliorate obesity-associated metabolic disorders via significantly decreasing hepatic SREBP-1 expression in Zucker diabetic fatty (ZDF) rats [33]. Our study also showed similar data with previous reports showing that FO+ALA-PS elevated mRNA and protein expressions of PPAR*α* and decreased SREBP-1, which may explain the lower concentrations of TG in FO+ALA-PS-treated group compared to HFD-treated group. Since the activations of PPAR*α* and SREBP-1c have been shown to stimulate FA beta-oxidation and TG synthesis, respectively [34, 35], we speculate that increased beta-oxidation of FA and reduced TG synthesis are likely responsible for the TG-lowering effect of FO+ALA-PS. Based on the important lipid modulation role of molecules mentioned above and the results in present study, we suggest that improvement effect of FO+ALA-PS on lipid profiles and atherosclerosis may be linked to regulate HMGCR, SREBPs, and PPAR*α*.

Besides hyperlipidemia, inflammation plays a crucial role in the etiology of atherosclerosis, from development of fatty streaks to plaque rupture and thrombosis. Once activated by stimuli such as oxidized lipoproteins, hypertension, or hyperglycemia, vascular endothelial cells express VCAM-1, which enhances the recruitment and adhesion of monocytes to the endothelium [36]. TNF-*α*, IL-6, and IL-1*β* increase VCAM-1 expression and mediate localization and recruitment of monocytes into the subendothelial space [37]. Chemoattractant, such as MCP-1, directly migrates leukocytes into the intima and recruits macrophage to the vessel wall [38]. Such endothelial injury ultimately leads to platelet aggregation and the release of platelet-derived growth factor, which initiates the proliferation of smooth muscle cells (SMCs) in the arterial intima, leading to the formation of atherosclerotic plaque [39]. Since these inflammatory markers have a key role in mediating inflammatory cascades and promoting atherosclerosis formation, the positive impact of dietary FO+ALA-PS on plasma levels, mRNA, and protein expressions of VCAM-1, TNF-*α*, IL-6, IL-1, and MCP-1 observed in this research supported that better function in atherosclerosis prevention of FO+ALA-PS is at least partly through perfecting inflammatory response. However, previous researches showed that consumption of PS esters of sunflower oil and fish oil had no impact on plasma TNF-*α*, IL-6, and C-reactive protein in hypercholesterolemic persons [40]. One possibility to explain this discrepancy might be that flaxseed oil supplemented with ALA-PS offers a better anti-inflammatory approach due to the large content of ALA in flaxseed oil because dietary consumption of ALA has been demonstrated to reduce circulating levels of several atherogenic and inflammatory markers, including C-reactive protein, serum amyloid A, IL-6, and soluble VCAM-1 in dyslipidemic patients [41, 42]. On the other hand, previous report showed that high levels of plasma n-3 fatty acids were independently associated with lower levels of proinflammatory markers (IL-6, IL-1ra, TNF*α*, and C-reactive protein) [43]. Thus, anti-inflammatory effects of FO+ALA-PS described in this study may be also associated with its high ratio of n-3 to n-6 fatty acids.

Furthermore, previous publication also pointed out that chronic and acute overproduction of ROS under pathophysiologic conditions was integral in the development of atherosclerosis [44]. In present research, a pronounced reduction of aorta ROS along with lower concentrate of MDA and higher level of GSH was observed in FO+ALA-PS-fed animals than those in mice on HFD. These results suggested that FO+ALA-PS-mediated prevention of atherosclerosis is closely related with inhibition of oxidative stress. Our data is similar with previous reports that flaxseed oil or PS treatment has an antioxidative function [45, 46]. However, the effects of FO+ALA-PS on oxidative stress and the underlying molecular mechanisms for which are very limitedly reported. In the context of cardiovascular disease, oxidative stress leads to an increased ROS production largely determined by NADPH oxidase, a multicomponent enzyme consisting of four major units: p22^phox^, gp91^phox^, p47^phox^, and p67^phox^ [47]. Some evidences showed that increased expression of p22^phox^ contributed to the process of atherosclerosis and apoE^−/−^ mice lacking p47^phox^ had a marked reduction of atherosclerosis in the descending aorta [48, 49]. Here, we demonstrated that FO+ALA-PS treatment reversed mRNA and protein expressions of p22^phox^, p47^phox^  p67^phox^, and gp91^phox^ in aorta. These data indicate a mechanism that FO+ALA-PS inhibits aortic NADPH oxidase for ROS reduction and results in protection effects of atherosclerosis.

## 5. Conclusions

Our data is supportive of the synergistic and complementary effects of flaxseed oil containing ALA-PS on overall lipid, systemic inflammation, and oxidative stress which result in further amelioration in atherosclerosis. This study may provide new insights that dietary flaxseed oil containing ALA-PS therapy may be an ideal alternative or adjunct to pharmacological treatment for maximum cardioprotection in high risk individuals and flaxseed oil might be a good source for plant sterol ester supplementation as well as a good candidate to replace other fats in functional foods.

## Figures and Tables

**Table 1:** 

Gene	Forward primer	Reverse primer
HMGCR	5′-AGCCGAAGCAGCACATGAT C-3′	5′-CTTGTGGAATGCCTTGTGATTG-3′
SREBP-2	5′-AAGTCTGGCGTTCTGAGGAA-3′	5′-GTTCTCCTGGCGAAGCT-3′
PPAR*α*	5′-GGAGTGCAGCCTCAGCCAAGTT-3′	5′-AGGCCACAGAGCGCTAAGCTGT-3′
SREBP-1c	5′-TCCTTAACGTGGGCCTAGTCCGAAG-3′	5′-GCTCGAGTAACCCAGCACGGG-3′
VCAM-1	5′-GATAGACAGCCCACTAAACG-3′	5′-CAATGACGGGAGTAAAGGT-3′
TNF-*α*	5′-TCTCATTCCTGCTTGTGG-3′	5′-ACTTGGTGGTTTGCTACG-3′
MCP-1	5′-GCAGGTGTCCCAAAGAA-3′	5′-GGTGGTTGTGGAAAAGG-3′
IL-6	5′-ATTTCCTCTGGTCTTCTGG-3′	5′-TGGTCTTGGTCCTTAGCC-3′
IL-1*β*	5′-CTTTCCCGTGGACCTT-3′	5′-ATCTCGGAGCCTGTAGTG-3′
p22^phox^	5′-GTGTGCGCAGGGTCCTCGTC-3′	5′-TCCAACCTGTGGCCGCTCCT-3′
p47^phox^	5′-GGTCGACCATCCGCAACGCA-3′	5′-GCGGCGATAGGTGTCCTGGC-3′
p67^phox^	5′-GTGGGTCGGCTGTTCCGTCC-3′	5′-GCGTCTCCGGCACAAAGCCA-3′
gp91^phox^	5′-GGGGCCTGTATGTGGCCGTG-3′	5′-AGGCATGCGTGTCCCTGCAC-3′

**Table 2:** Effects of FO+ALA-PS on lipid profiles in mice.

	Groups			
	Control	HFD	FO	FO+ALA-PS
Serum TC (mmol/L)	1.72 ± 0.24	20.77 ± 5.00^a^	16.04 ± 4.59^a^	11.33 ± 3.15^abc^
Serum TG (mmol/L)	0.91 ± 0.15	2.27 ± 0.40^a^	1.83 ± 0.42^ab^	1.39 ± 0.41^abc^
Serum LDL-C (mmol/L)	0.31 ± 0.12	12.16 ± 3.11^a^	10.24 ± 2.32^a^	4.17 ± 1.35^abc^
Serum HDL-C (mmol/L)	1.01 ± 0.14	0.26 ± 0.12^a^	0.45 ± 0.23^ab^	0.60 ± 0.16^abc^
Liver TC (mg/g)	2.54 ± 0.19	4.09 ± 0.75^a^	3.52 ± 0.38^a^	2.55 ± 0.24^bc^
Liver TG (mg/g)	26.47 ± 6.40	64.04 ± 18.49^a^	40.81 ± 7.63^ab^	30.80 ± 6.21^bc^

Forty-two apoE KO mice were randomly divided into 3 groups on average, namely, HFD, FO, and FO+ALA-PS. Fourteen wild type C57BL/6 mice were divided into control group. Values are given as means ± SD (*n* = 14). ^a^ *P* < 0.05 versus the control; ^b^ *P* < 0.05 versus the HFD group; ^c^ *P* < 0.05 versus the FO group. HFD: high fat diet; FO: HFD + 5% flaxseed oil; FO + ALA-PS: HFD + 2.7% flaxseed oil + 3.3% flaxseed oil ester of plant sterol.
